# Genome-Wide Identification of MAPKK and MAPKKK Gene Family Members and Transcriptional Profiling Analysis during Bud Dormancy in Pear (*Pyrus x bretschneideri*)

**DOI:** 10.3390/plants11131731

**Published:** 2022-06-29

**Authors:** Qin Liang, Xiaojie Lin, Jinhang Liu, Yu Feng, Xianqian Niu, Chao Wang, Keke Song, Chao Yang, Liang Li, Yongyu Li

**Affiliations:** 1 Institute of Natural Products of Horticultural Plants, Fujian Agriculture and Forestry University, Fuzhou 350002, China; liangqin0803@163.com (Q.L.); 18959187291@163.com (X.L.); liujinhang710@163.com (J.L.); fengyu12234567@163.com (Y.F.); wangchao1551676@163.com (C.W.); skk187375@163.com (K.S.); yycc32@126.com (C.Y.); 2 Guang’an Modern Agricultural Industrial Park Service Center, Guangan 638500, China; 3 Fujian Institute of Tropical Crops, Zhangzhou 363001, China; nxq828@126.com; 4 Fruit Research Institute, Fujian Academy of Agricultural Sciences, Fuzhou 350002, China

**Keywords:** *Pyrus*, bud dormancy, MAPK member-mediated genes, gene family, bioinformatics analysis

## Abstract

The mitogen-activated protein kinase (MAPK) cascade consisting of three types of reversibly major signal transduction module (MAPKKK, MAPKK, and MAPK) is distributed in eukaryotes. MAPK cascades participate in various aspects of plant development, including hormone responses, cell division and plant dormancy. Pear is one of the most economically important species worldwide, and its yield is directly affected by dormancy. In this study, genome-wide identification of MAPKK and MAPKKK gene family members in *Pyrus x bretschneideri* and transcriptional expression analysis of MAPK cascades during pear dormancy were performed. We identified 8 MAPKKs (PbrMKKs) and 100 MAPKKKs (PbrMAPKKKs) in *Pyrus* using recent genomic information. PbrMAPKKs were classified into four subgroups based on phylogenetic analysis, whereas PbrMAPKKKs were grouped into 3 subfamilies (MEKK, Raf, and ZIK). Most PbrMAPKKKs and PbrMAPKKs in the same subfamily had similar gene structures and conserved motifs. The genes were found on all 17 chromosomes. The comprehensive transcriptome analysis and quantitative real-time polymerase chain reaction (qRT–PCR) results showed that numerous MAPK cascade genes participated in pear bud dormancy. The interaction network and co-expression analyses indicated the crucial roles of the MAPK member-mediated network in pear bud dormancy. Overall, this study advances our understanding of the intricate transcriptional control of MAPKKK-MAPKK-MAPK genes and provides useful information on the functions of dormancy in perennial fruit trees.

## 1. Introduction

Pear, a bulk fruit tree, is the main trade fruit worldwide. In China, it is one of the three major fruits in terms of output, along with apple and citrus. Pear is a typical deciduous tree: bud dormancy occurs in winter to avoid adverse growth environments and to maintain the ability to continue to grow and develop [1]. Due to global warming, pear faces the problem of insufficient chilling requirements for breaking dormancy and flowering, and the development of pears suitable for the growth conditions in South China is limited. In addition, the mechanism underlying pear dormancy in winter is still very unclear, which restricts the development of the pear industry.

Bud dormancy is an adaptation to environmental and seasonal changes acquired by plants through long-term evolution. Once the bud enters internal dormancy (endodormancy), its growth is inhibited by internal conditions, and the bud cannot resume growth until the cooling requirement, which is genetically determined, is met. After internal dormancy, the bud gradually shifts to ecological dormancy (ecodormancy), where dormancy is triggered by environmental factors and the bud can resume growth only under appropriate conditions [2]. Inappropriate conditions will hinder the next stage of growth and development and result in flower organ malformations or serious abortive events, which affect the yield and quality of the fruit. Studies have found that flower bud dormancy is associated with temperature, hormones, and active oxygen [3,4].

The mitogen-activated protein kinase (MAPK) pathway has been recognized as a highly conserved signal transduction pathway among all eukaryotes that regulates numerous cellular processes, such as cell division, developmental programmes, hormonal responses, and biotic and abiotic stress responses [5,6,7]. This pathway mainly involves a linear cascade of three consecutively acting protein kinases: MAPKK kinases (MAPKKKs), MAPK kinases (MAPKKs), and MAP kinases (MAPKs) [8]. They perform their functions through sequential phosphorylation. Upstream signals first activate MAPKKKs, which in turn activate MAPKKs, and then specific MAPKs are activated by MAPKKs [9]. MAPKKs are activated when serine and serine/threonine residues in the S/TXXXXXS/T motif are phosphorylated by MAPKKKs [10]. The three classes of MAPK cascade genes represent different gene families and are encoded by multiple genes [11]. To date, extensive studies have been conducted to systematically investigate the three gene families in many plant species, and 20 MAPK, 10 MAPKK, and 80 MAPKKK genes have been reported in the *Arabidopsis* genome [8,12,13], whereas the rice genome contains 17 MAPK, 8 MAPKK, and 75 MAPKKK genes [14,15]. In recent studies on apple, 26 putative MAPK genes and 9 putative MAPKK genes have been discovered [16]. In addition, the Chinese white pear genome includes 23 MAPK genes [17].

In plants, the most extensively studied MAPKs are AtMPK3/4/6 in *Arabidopsis* and other plant species. The MKK2-MPK4/MPK6 cascade has been shown to be activated by cold and salt stresses [13]. AtMKK1 mediates abscisic acid (ABA)-induced CAT1 expression and H_2_O_2_ production via AtMPK6-coupled signalling in *Arabidopsis* [18]. More recently, it has been reported that the MEKK1–MKK2–MPK4/MPK6 cascade is involved in cold and salt stress signalling in *Arabidopsis* [13]. ANP2/3-MKK6-MPK4/11/13 play roles in the regulation of cytokinesis [19,20]. These results indicate that the MAPK cascade is strongly associated with dormancy. Du et al. [21] speculated that MAPK genes might be involved in the regulation of dormancy release in flower buds. Furthermore, the MKKK62-MKK3-MAPK7/14 module negatively regulates seed dormancy in rice [22].

We previously reported that MAPK family members were significantly differentially expressed during pear dormancy. Therefore, we speculated that the MAPK cascade coordinates to regulate bud break and dormancy release in pear. However, the MAPKK and MAPKKK gene families have not been identified, and the mechanism of their dormancy regulation in plants is unclear. In this study, we identified 8 MAPKKs and 100 MAPKKKs at the genome-wide level of pear and systematically analysed their phylogenetic relationships, gene structure, conserved motifs and chromosomal location. Subsequently, we used RNA sequencing (RNA-Seq) and quantitative real-time polymerase chain reaction (qRT–PCR) to investigate the expression patterns of these genes in different stages of bud dormancy in pear. The purpose of this study was to identify MAPKKs and MAPKKKs in pear and to conduct a preliminary study on their expression during flower bud dormancy.

## 2. Results

### 2.1. Identification and Sequence Analysis of Pyrus MAPKKs and MAPKKKs

The release of complete *Pyrus x bretschneideri* genome sequences has made it possible to identify all MAPKK and MAPKKK family members in this plant species for the first time [23]. Our genome-wide analysis revealed 8 PbrMAPKKs and 100 PbrMAPKKKs (including 24 MEKKs, 57 Rafs, and 19 ZIKs). The newly identified putative MAPKK and MAPKKK genes were designated sequentially based on their distribution on chromosomes [24]. The gene length for 8 PbrMAPKKs ranged from 951 bp (PbrMKK3) to 1872 bp (PbrMKK7), and the genes encoded proteins ranging from 316 to 519 amino acids (aa) in length, with an MW of 34.8 (PbrMKK3)–57.8 (PbrMKK7) kDa and a pI of 5.52 (PbrMKK7)–9.15 (PbrMKK8) (Table S2). The gene length of 100 predicted PbrMAPKKKs ranged from 1267 bp (PbrZIK18) to 4894 bp (PbrRaf16), and the genes encoded proteins ranging from 295 to 1421 aa in length, with an MW of 33.8–154.4 kDa and a pI of 4.58 (PbrMEKK5)–9.46 (PbrRaf1) (Table S3).

### 2.2. Phylogenetic Analysis and Multiple Alignment of MAPKK and MAPKKK Genes in Pyrus

To investigate the evolutionary relationships of the MAPKK and MAPKKK families in *Pyrus* with those in *Arabidopsis* and *Malus*, NJ trees were constructed with MAPKK and MAPKKK proteins. Then, conserved kinase domains were aligned using ClustalX 1.8. Based on the resulting phylogenetic tree, the MAPKK proteins were divided into four groups, A, B, C and D (Figure S1). In comparison with those in other plants (*Arabidopsis*, rice and *Malus*) [9,25,26], the MAPKK gene family in pear is highly conserved. The MAPKKK family forms the largest group of MAPK pathway components and is grouped into 3 clusters, named the MEKK, ZIK and Raf subfamilies (Figure S2). The Raf subfamily is the largest, similar to findings in other plants. According to multiple sequence alignment results, MAPKK contains conserved motifs of the activation loop (-S/TxxxxxS/T- and -VGTxxYMSPER-) and is the phosphorylated object of MAPKKKs, and the active site (-D(L/I/V)L- or -K/R-K/R-K/RxxxxxL/IxL/I-) performs MAPK phosphorylation (Figure S3). All the MEKK, Raf, and ZIK subfamilies in *Pyrus* have conserved signatures of -G(T/S)Px(W/Y/F)MAPEV-GTxx(W/Y)MAPE- and -GTPEEMAPE(L/V/M)(Y/F/L)-, which are similar to those of the MAPKKKs in *Arabidopsis* and other plant species [27] (Figure S4).

### 2.3. Gene Structure, Conserved Motifs and Chromosomal Distribution Analysis of Pyrus MAPKKs and MAPKKKs

Exon-intron structures of the MAPKK and MAPKKK families were also investigated to gain insight into the structural evolution of these genes. In the MAPKK family, group A (PbrMKK1, PbrMKK5, and PbrMKK7) has the most exons, with more than 8, whereas the others have fewer than 2 exons. Group B (PbrMKK3) without the 3′UTR and 5′UTR did not have introns (Figure S5B). In the MAPKKK family, genes in the MEKK subfamily contained the fewest exons (fewer than 2), followed by ZIK, while most Raf genes had more than 6 exons (Figure S6). These results demonstrated that MAPKKK and MAPKK members in the same group have similar gene structures, which may be correlated with gene evolution. To determine the evolution of proteins in these two families, the 20 conserved motifs in each of the two protein families were identified with Multiple Em for Motif Elicitation (MEME). Using the results from SMART and Pfam analysis, we found that all the identified PbrMAPKKs and PbrMAPKKKs contained the protein kinase domain. For the MAPKK family, the protein kinase domain of motifs 1–3 was located in the middle of the protein (Figure S5). For the MAPKKK family, the protein kinase domains of motif 2 and motif 3 were located in the middle of the protein (Figure S7).

**Figure 1:**
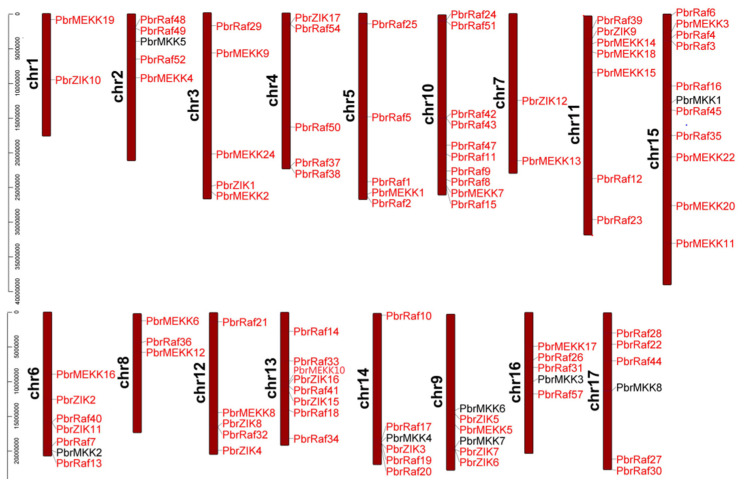
Genomic distribution of PbrMKKs and PbrMAPKKKs on *Pyrus* chromosomes. The chromosome number is indicated at the left of each chromosome representation. The scale is in base pair (bp).

The position and transcriptional direction of each gene are shown in Figure 1, and accurate positions on the *Pyrus* chromosomes are provided in Tables S2 and S3. The eight MAPKK gene families were distributed on eight different chromosomes, while the MAPKKK gene families were distributed on all 17 chromosomes, and the analysis also revealed that one particular gene replication event occurred on chromosomes 2, 3, 4, 5, 6, 9, 10, 11, 12, 13, 14, 15, and 16. In addition, some genes were positioned at the ends of chromosomes. For example, PbrRaf30 was located at the bottom of chromosome 17, and PbrMEKK19 was located at the top of chromosome 1.

### 2.4. Expression Profiles of MAPK Cascade Genes in Different Stages of Dormancy in Pear

**Figure 2:**
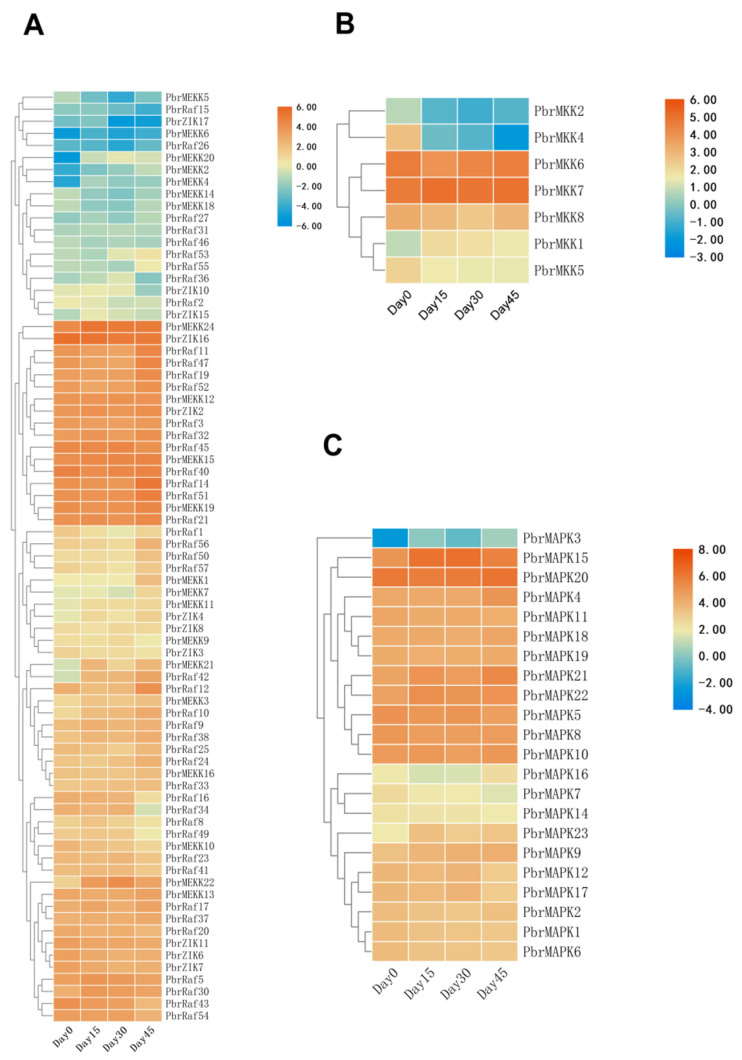
Hierarchical clustering of differentially expressed MAPK cascade genes in different stage of dormancy. (**A**) The PbrMAPKKKs expression. (**B**) The PbrMKKs expression. (**C**) The PbrMAPKs expression. Dormancy-specific expression levels of ‘Huanghua’ pear MAPK cascade genes were obtained from the RNA-Seq data (SRA accession number: PRJNA587390). The colour represents MAPK cascade genes expression levels: Log2 (FPKM). The phylogenetic relationship was shown on the left.

The gene expression database of pear (‘Huanghua’) at different dormancy stages from the NCBI SRA (accession number: PRJNA587390) was used. Our previous experiments showed that 0-cold-day buds (Day 0) did not enter dormancy, 15-cold-day (Day 15) and 30-cold-day buds (Day 30) were in endodormancy, and 45-cold-day buds (Day 45) were released from endodormancy. Generally, the majority of MAPK cascade genes were broadly expressed at different dormancy stages (0, 15, 30, and 45 days after flowering (DAF)) of pear, with variations between specific individuals (Figure 2). Of all the genes studied, only two (PbrMEKK17 and PbrMEKK23) in the MEKK subfamily were not expressed, and only one in the MAPK family (PbrMAPK13) was not expressed. For the Raf and ZIK subfamilies, 45/57 and 13/19 of genes were expressed in different dormancy stages, respectively. To identify MAPK cascade genes associated with the dormancy process in pear, two libraries were compared with each other, using |DESeq2log2(FC)≥1 and padj < 0.05 as the screening criteria; the differential expression from Day 0 to Day 15 and Day 30 to Day 45 was significantly improved. All differential expression levels of the MAPK cascade genes among all samples are shown in Table 1, which suggest that MAPK cascade genes are involved in the regulation of flower bud dormancy in pear, especially the processes of entering dormancy and breaking dormancy.

### 2.5. MAPK Cascade Networks and Their Co-Expression during Bud Dormancy

**Figure 3:**
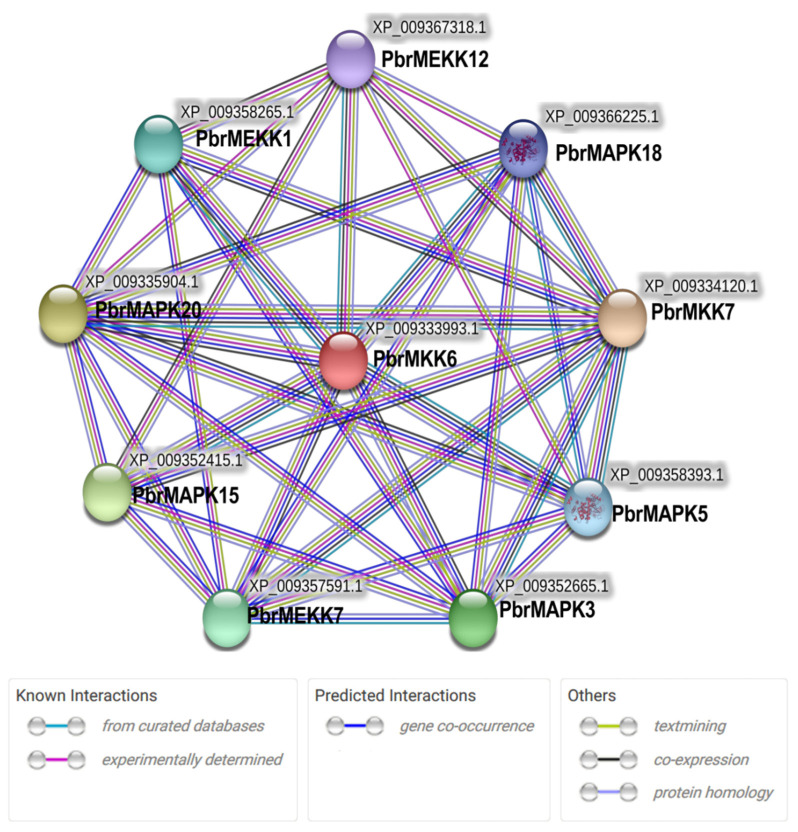
Interaction network and co-expression analyses of *Pyrus* PbrMAPKKK-PbrMAPKK-PbrMAPK cascade. The circles in the figure are nodes. Each node represents a protein. The helix in the protein indicates that the structure of the protein is known. The lines between nodes represent interactions between two proteins, and different colours represent different types of interactions.

To better understand the function of MAPKKK-MAPKK-MAPK signalling cascades in dormancy, the interaction networks and co-expression of 9 MAPK cascade genes were investigated based on experimentally validated interactions and transcriptome data. A *Pyrus* MAPKK-mediated interaction network was created, and 9 interactive proteins (with high confidence; score > 0.87) were identified with the STRING database (upstream PbrMEKK1, PbrMEKK7, and PbrMEKK7 genes and downstream PbrMAPK15 and PbrMAPK18 genes) (Figure 3). According to the STRING database, the proteins have an interactive relationship. Notably, PbrMKK6 and PbrMKK7 exhibited relatively high transcript abundances during dormancy, and their upstream genes (PbrMEKK1, PbrMEKK7, and PbrMEKK12) and downstream genes (PbrMAPK3, PbrMAPK5, PbrMAPK15, and PbrMAPK20) exhibited considerable variation in expression levels (Figure 2). Collectively, the interaction network and co-expression results indicated the crucial roles of the MAPK member-mediated network in pear bud dormancy.

### 2.6. Validation of PbrMAPKKK-PbrMAPKK-PbrMAPK Cascade Gene Function in Pear Dormancy by qRT–PCR Analysis

**Figure 4:**
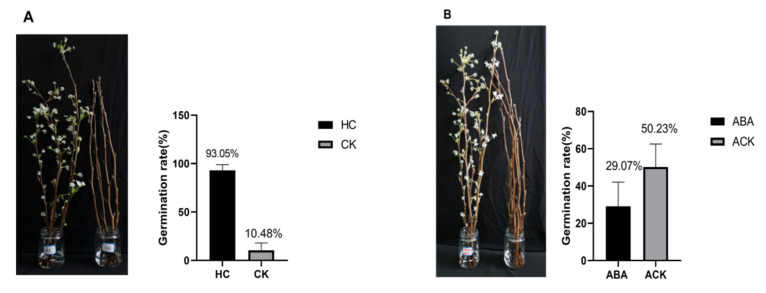
(**A**) Germination results response to 1% HC treatment. Comparison of germination results, the left side is the HC treatment group and the right side is the control group; (**B**) Germination results response to 150 mg/L ABA treatment. Comparison of germination results, the right side is the 150 mg/L ABA treatment group and the left side is the control group.

**Figure 5:**
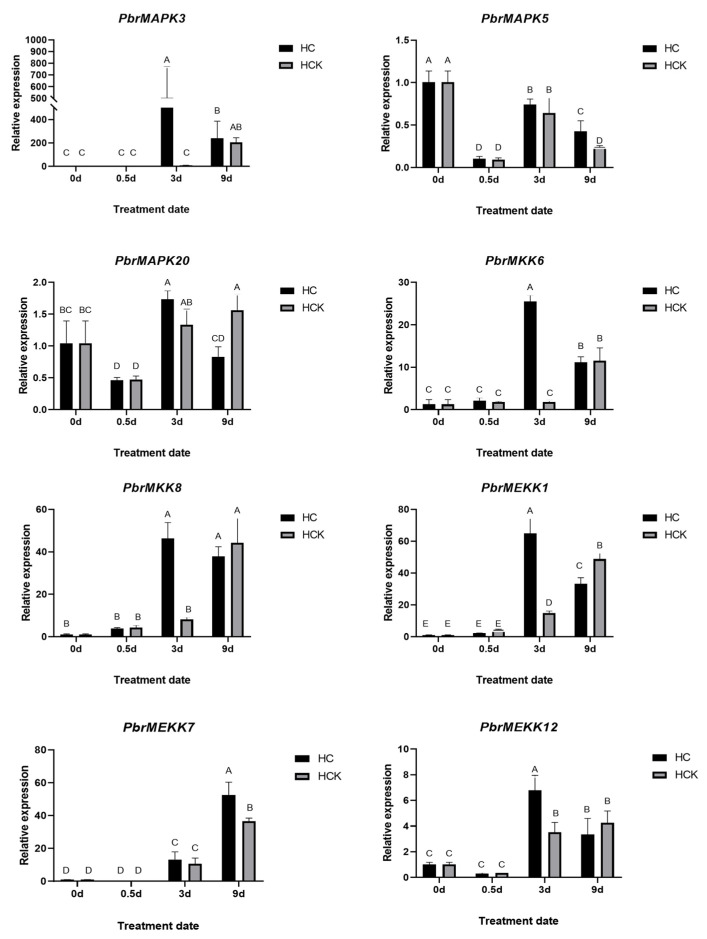
Differential expression for 8 MAPK cascade genes response to 1% HC treatment at 0 day, 0.5 day, 3 day, and 9 day, with water treatment as control. The above letter ABCDE is the significant difference analysis result after graphPad analysis.

**Figure 6:**
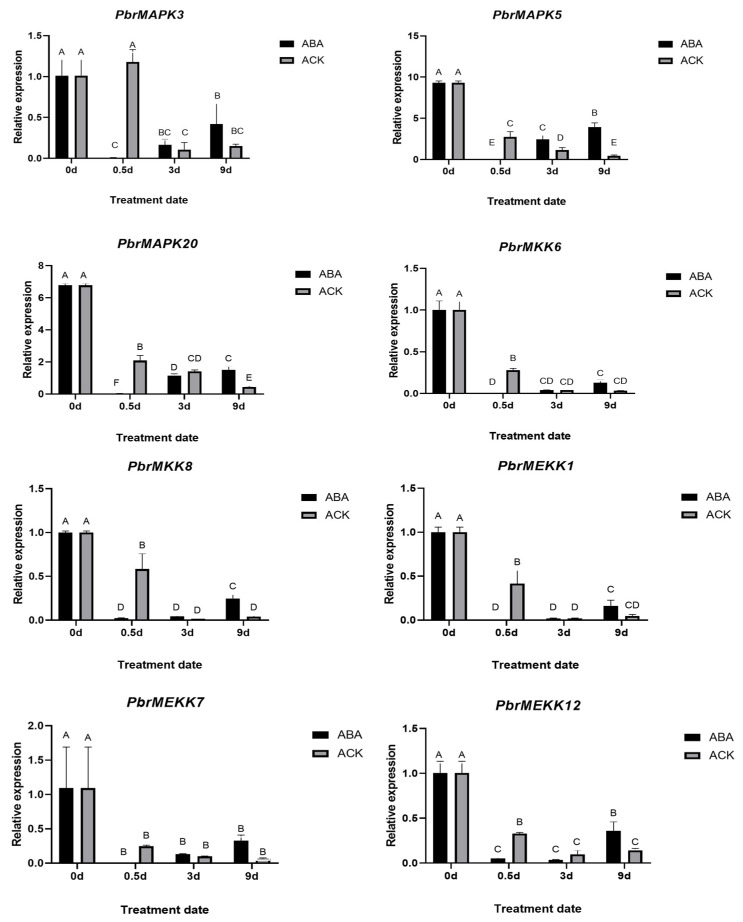
Differential expression for 8 MAPK cascade genes response to ABA treatment at 0 day, 0.5 day, 3 day, and 9 day, with water treatment as control. The above letter ABCDEF is the significant difference analysis result after graphPad analysis.

To verify that these genes are involved in the pear dormancy process, 1% HC was used artificially to break bud dormancy, and 150 mg/L ABA was used to induce bud dormancy. After 21 days of treatment, a bud break rate less than 50% was considered to indicate retention in the internal dormancy stage, and a rate greater than 50% was considered to indicate a lack of function [28]. The bud break rate of the 1% HC treatment group was 93.2%, while that of the control group was 10.48% (Figure 4A), which indicated that buds in the treated group were released from dormancy, while those in the control group were still dormant. Similarly, buds in the 150 mg/L ABA-treated group broke, and those in the control group exhibited dormancy (Figure 4B). Compared with those in the control, all the cascade genes in the 1% HC group had high expression after 3 days of treatment (Figure 5). Interestingly, expression of the cascade genes was barely detectable or low in the 150 mg/L ABA-treated group compared with the corresponding control sample after 0.5 days (Figure 6). This finding demonstrated the role of the MAPK cascade in regulating the dormancy of pear buds and that the cascade is very likely to regulate entrance into dormancy.

## 3. Discussion

MAPK cascades act as important signal transduction modules in the regulation of plant developmental activities and responses to the environment [29]. An MAPK cascade, in its simplest form, consists of an MAPKKK-MAPKK-MAPK module that is linked in various ways to upstream receptors and downstream targets [8]. Through a genome-wide search using the latest available *Pyrus x bretschneideri* information, a total of 8 putative PbrMAPKKs and 100 putative PbrMAPKKKs were obtained, which were classified into 4 and 3 subgroups, respectively, according to their phylogenetic relationships. This classification is consistent with that of the MAPKK and MAPKKK families in other plants [8,12,29]. The phylogenetic relationships, conserved protein motifs and exon/intron organization strongly supported the identity of each subgroup. Analysis of conserved motifs in these two families revealed that -D(I/L/V)K-, -S/T-X5-S/T- and -VGT-X2-YMSPER- form the MAPKK kinase domain. MEKK (-G(T/S)Px(W/Y/F)MAPEV-),Raf(-GTxx(W/Y)MAPE-), and ZIK(-GTPEEMAPE(L/V/M)(Y/F/L)-) indicated that -MAPE- are the core amino acid residues within the MAPKKK kinase domain. These typical characteristics of the two families were also observed in other plants, such as *Jatropha curcas,* rice and cucumber [25,27,30]. Conserved motifs and exon–intron organization suggested that PbrMAPKKs and PbrMAPKKKs in the same group had a close relationship during gene evolution.

Pears are the second most productive fruit after apples. Early-ripening pear (in which maturity occurs before early August) is popular in southern China because of its market availability in July and August when there are few other pears [31] After chilling requirements are met, floral buds shift from being endodormant to being ecodormant and regain their flowering ability [32]. Dormancy affects the fruit yield and quality of pear in South China because low temperature accumulation is insufficient in winter [33]. In this study, we found that more than 60.0% of MAPK cascade genes were expressed during bud dormancy and established their network and co-expression patterns in *Pyrus*, thus revealing the relationship of the MAPK cascade with bud dormancy. This function has also been reported in nectarines [21]. In addition, we verified gene expression by using HC and ABA to artificially release dormancy and induce dormancy, respectively. We found that PbrMAPKKs (PbrMKK6 and PbrMKK8) and PbrMAPKKKs (PbrMEKK1, PbrMEKK7, and PbrMEKK12) showed relatively high expression during dormancy breakage. They may interact and have a coregulatory effect during dormancy.

**Figure 7:**
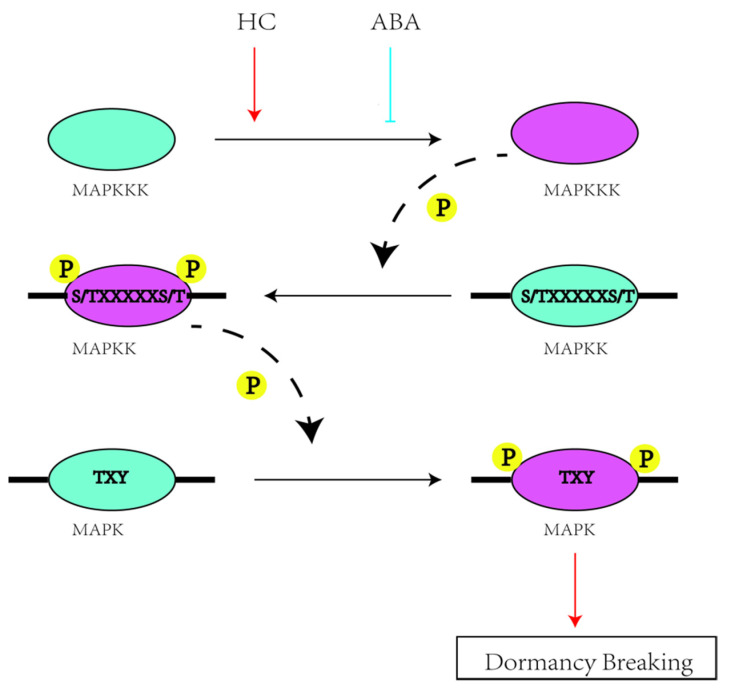
Possible pathways of MAPK cascade genes involved in the dormancy breaking in pear. Red lines with arrows denote a positive effect with an indirect interaction; Blue lines with T-ends indicate the inhibition through genetic interaction; Black dotted lines indicate phosphorylation. Blue ellipse represents an inactive state purple ellipse indicates the active state.

Multiple studies have examined the involvement of the MAPK cascade in cold stress and hormone responses. Cold stress activates the AtMEKK1-AtMKK1/2-AtMPK453 and AtMPK6-p44MAPK cascades in *Arabidopsis thaliana* [34]. Moreover, the upregulation of MAPK cascade genes under cold stress has been demonstrated in numerous plants, including CsMPK3, CsMPK7, and CsMPK13 in cucumber [30] and JcMAPK4, JcMAPKK5, and eight JcMAPKKKs in *Jatropha curcas* [27]. MAPKs are hormone receptors, and recent evidence shows that the complete MAP3K17/18-MKK3-MPK1/2/7/14 module is under the control of ABA [35]. GHMKK4 increased sensitivity to ABA and gibberellin A3 (GA3) in Nicotiana benthamiana [36]. Interestingly, pear dormancy is closely related to low temperature demand and hormones [33,37,38]. Synergy between the MAPK cascade pathway and ABA signal transduction is involved in the regulation of seed germination [39]. Our previous studies have shown that ABA promotes pear dormancy [40], but a connection between the MAPK cascade pathway and ABA has not been reported. Here, we constructed a *Pyrus* MAPKK-mediated interaction network with 8 interactive proteins by using the STRING database, and their expression patterns were confirmed by qRT–PCR. Pérez’s experiments with grapevine buds showed that the expression and activity of catalase were inhibited by HC, and H_2_O_2_ enhancement has been associated with the breakage of endodormancy [41]. Reactive oxygen species (ROS) may activate MAPK pathways via inhibition and/or degradation of MAPKs [42]. ABA-induced stomatal closure requires H_2_O_2_, and NADPH oxidase is the main source of H_2_O_2_ [43]. A mutation in the gene encoding the catalytic subunit of NADPH oxidase leads to the loss of ABA-induced ROS production [44]. MAPKs have also been implicated in ABA signalling, which was discovered long before MAPK activity was observed in guard cell protoplasts after treatment with ABA [45]. Based on the above studies, we speculated that ABA inhibited MAPK cascade pathway-mediated ROS during endodormancy in pear buds. MAPKKKs sense external stimuli and activate MAPKKs through phosphorylation; HC promoted activation, while ABA inhibited it (Figure 7). This may be related to the dephosphorylation effect of ABA [46]. Then, the phosphorylated MAPKKs activate MAPKs through phosphorylation. Activated MAPKs have a certain promoting effect on pear flower bud dormancy breaking. The specific regulatory mechanism needs further study.

## 4. Materials and Methods

### 4.1. Identification of the MAPKK and MAPKKK Gene Families in Pyrus

Methods of genetic identification followed those of Rao et al. [25] and additional optimizations. Whole pear (*Pyrus x bretschneideri*) protein sequences were acquired from the NCBI genome database. To identify members of the MAPKK and MAPKKK gene families, hidden Markov model (HMM) profiles were built with MAPKK and MAPKKK proteins from *Arabidopsis* and rice. HMMER software was used to search for predicted homologous proteins in the *Pyrus* dataset [47]. Genes with e-values less than 1e-10 were selected as candidate genes for the MAPKK and MAPKKK gene families of *Pyrus*. Then, filtering was carried out using BLASTP, with 50% identity used as the threshold for the sequences obtained from BLAST analysis. All filtered protein sequences derived from the candidate genes collected were examined using the domain analysis programs Pfam (Protein family: http://pfam.sanger.ac.uk/ (accessed on 19 May 2022)) and SMART (http://smart.embl-heidelberg.de/ (accessed on 19 May 2022)) to confirm that the predicted *Pyrus* MAPKK and MAPKKK proteins contained the domain for protein kinases [47].

### 4.2. Multiple Sequence Alignments and Phylogenetic Analysis

Multiple sequence alignments of putative MAPKK and MAPKKK sequences were performed using ClustalX 1.8 (http://www.clustal.org/clustal2/ (accessed on 19 May 2022)), and then a phylogenetic tree was constructed using the neighbour-joining (NJ) method in Molecular Evolutionary Genetics Analysis (MEGAX) 32 software (https://www.megasoftware.net/ (accessed on 19 May 2022)).

### 4.3. Sequence, Protein Property and Chromosome Location Analyses

The characteristics of PbrMPKKs and PbrMPKKKs, including protein length, isoelectric point (pI), and molecular weight (MW), were predicted by the ExPASy Proteomics Server (https://web.expasy.org/compute_pi/ (accessed on 19 May 2022)). The novel motifs of PbrMAPKKs and PbrMAPKKKs were searched using MEME 5.0.3 (http://meme-suite.org/tools/meme (accessed on 19 May 2022)) [48]. The parameters were set as follows: the site distribution was set to any number of repetitions (anr), the number of motifs was set to 20, the width of the motif was limited to between 10 and 30, and other optional parameters remained as the defaults [49]. The GSDS database (http://gsds.cbi.pku.edu.cn/ (accessed on 19 May 2022)) was used to analyse gene structure. The physical positions of the MAPKK and MAPKKK genes were obtained from the *Pyrus* annotation project database of the Genome Database for *Rosaceae* (GDR).

### 4.4. Differential Expression Analysis of MAPK Cascade Genes during Dormancy Using RNA-Seq

RNA-Seq data collected during dormancy were obtained from our previous studies (NCBI Sequence Read Archive (SRA) accession number: PRJNA587390) [50], and the transcript levels were determined as fragments per kilobase of exon per million fragments mapped (FPKM) values. The FPKM values are downloaded from the database and normalized. DESeq2 in R version 4.0.0 was used to detect differential expression between the sample groups.|DESeq2log2(FC)|≥1 refers to the ratio of expression levels between two samples (groups).

### 4.5. Plant Materials and Treatments

Hours of chilling are required for deciduous trees to break dormancy. The temperature limit is usually 7.2 °C [51]. Experimental materials (‘Huanghua’ pear) were collected from Jianning County, Sanming City, Fujian Province, to detect transcriptional changes in MAPK cascade genes during dormancy. Flower bud break can be advanced by spraying 2% hydrogen cyanamide (HC) in the dormancy period [52]. Branches of pear in endodormancy were taken from mother plants on December 1st, and 1% HC was sprayed onto the branches. Then, branches were cultivated under normal growth conditions (12 h/25 °C day and 12 h/25 °C night regime, and a relative humidity of 65%). Then, 0.5-day-old, 3-day-old and 9-day-old buds were collected from the branches. The same batch of branches was removed from 4 refrigeration houses (meeting their chilling hour requirement to break dormancy) and sprayed with 150 mg/L ABA on 8 January. Then, 0.5-day-old, 3-day-old and 9-day-old buds were collected from the branches.

### 4.6. MAPK Signalling Cascade Co-Expression Analysis during Pear Dormancy

To better understand the function of MAPKKK-MAPKK-MAPK signalling cascades in pear dormancy, interaction networks of MAPK cascade members in *Pyrus* from the STRING database (https://string-db.org/ (accessed on 19 May 2022)) were used, and co-expression was investigated based on validated experimental and transcriptomic data.

### 4.7. RNA Isolation and qRT–PCR Analysis

200 ng total RNA was isolated from plant samples using the RNAprep Pure Plant Plus Kit (TransGen Biotech Co., Ltd., Beijing, China). The extracted RNA was measured by spectrophotometer 260/280, and RNAs with OD value between 1.8–2.0 were selected for reverse transcription. 200 ng of total RNA was reverse-transcribed using TransScript One-Step gDNA Removal and cDNA Synthesis SuperMix (TransGen Biotech Co., Ltd., Beijing, China) in a final volume of 100 µL, Table 2 is the procedure of real-time PCR. All primers were designed by Primer5 software and synthesized commercially (Sangon Biotech Co., Ltd., Shanghai, China) (Table S1). The PpActin gene served as an internal control to normalize the expression levels [23]. qRT–PCR (TransGen Biotech Co., Ltd., Beijing, China) was performed using the LightCycler^®^96 quantitative PCR platform. To be sure of the absence of primer dimers or non-specific products, all samples we performed a Melting curve analysis from a real time PCR assay, and the experiments were independently repeated three times. Then relative expression levels were measured using the 2−ΔΔCt method [53]. The variance analysis was shown in Tables S4 and S5.

## 5. Conclusions

In conclusion, genome-wide analyses revealed 8 MAPKK and 100 MAPKKK genes in *Pyrus*. Expression profile analyses of MAPK cascade genes revealed their involvement in bud dormancy. Furthermore, interaction networks and co-expression analyses demonstrated the strong transcriptional response of MAPKKK-MAPKK-MAPK signalling in relation to dormancy regulation. In this case, qRT–PCR results also confirmed the synergistic involvement of MAPK Cascade genes in the dormancy regulation of pear buds. These data supply valuable information for the functional characterization of MAPKKK-MAPKK-MAPK genes and will serve as a basis for studies of bud dormancy.

## Figures and Tables

**Table 1:** Summary of differentially expressed MAPK cascade genes.

Differentially Expressed Gene Set	Differentially Expressed Gene Number	Up Regulated	Down Regulated
Day 0 vs. Day 15	66	32	34
Day 15 vs. Day 30	22	9	13
Day 30 vs. Day 45	66	47	19

**Table 2:** The procedure of real-time PCR.

Process	Temperature	Time	Cycle Number
Initial Denaturation	94 °C	30 s	1
Denaturation	94 °C	5 s	40
Annealing	60 °C	30 s	
Extension/Elongation	72 °C	10 s	

## Data Availability

Not applicable.

## Supplementary Materials

The following supporting information can be downloaded at: https://www.mdpi.com/article/10.3390/plants11131731/s1, Figure S1: Phylogenetic analysis of MAPKKs from *Pyrus*, *Malus*, *Arabidopsis*. The Neighbor-joining (NJ) tree was constructed using ClustalX 1.8 and MEGAX32 software. Figure S2: Phylogenetic analysis of MAPKKKs from *Pyrus* and *Arabidopsis*. The Neighbor-joining (NJ) tree was constructed using ClustalX 1.8 and MEGAX32 software. Figure S3: Multiple sequence alignment analysis of the peptides of MAPKK in *Pyrus*. The highlighted part showed the conserved signature motif obtained with the ClustalX program. Figure S4: Multiple sequence alignment analysis of the peptides of MAPKKK proteins in *Pyrus*. The highlighted part showed the conserved signature motif obtained with the ClustalX program. Figure S5: (A) Conserved motifs of PbrMAPKKs. All motifs were identified by MEME database. (B) Phylogenetic relationship and exon/intron structure of PbrMAPKKs. Gene structures were drawn using GSDS database. The blue boxes, green boxes, and the black lines indicate upstream/downstream, exons and introns, respectively. Figure S6: Phylogenetic relationship and exon/intron structure of PbrMAPKKs in *Pyrus*. For other details, see Figure S5. Figure S7: Conserved motifs of PbrMAPKKK family. All motifs were identified by MEME database. Table S1: Sequences of primers used in qRT–PCR. Table S2: Characteristics of the MAPKK genes in *Pyrus*. GeneID is accession number in NCBI database. Length (number of amino acids), molecular weight (kilodaltons), and isoelectric point (pI) of the deduced polypeptides were calculated using ExPASy (http://web.expasy.org/protparam/ (accessed on 19 May 2022)). Table S3: Characteristics of the MAPKKK genes in *Pyrus*. Genes have not been identified on chromosomes are presented scaffold sites. Table S4: Analysis of variance of 8 MAPK cascade genes response to 1% HC treatment at 0 day, 0.5 day, 3 day, and 9 day. Table S5: Analysis of variance of 8 MAPK cascade genes response to ABA treatment at 0 day, 0.5 day, 3 day, and 9 day.
