# Supplementary material for: Genome-Wide Identification of MAPKK and MAPKKK Gene Family Members and Transcriptional Profiling Analysis during Bud Dormancy in Pear (Pyrus x bretschneideri)
Source: Plants (Basel). 2022 Jun 29;11(13):1731. doi: 10.3390/plants11131731 (PMC9269224; doi:10.3390/plants11131731)
Supplement: Supplementary file 1 [file plants-11-01731-s001.zip › Supplementary Figures.pdf]

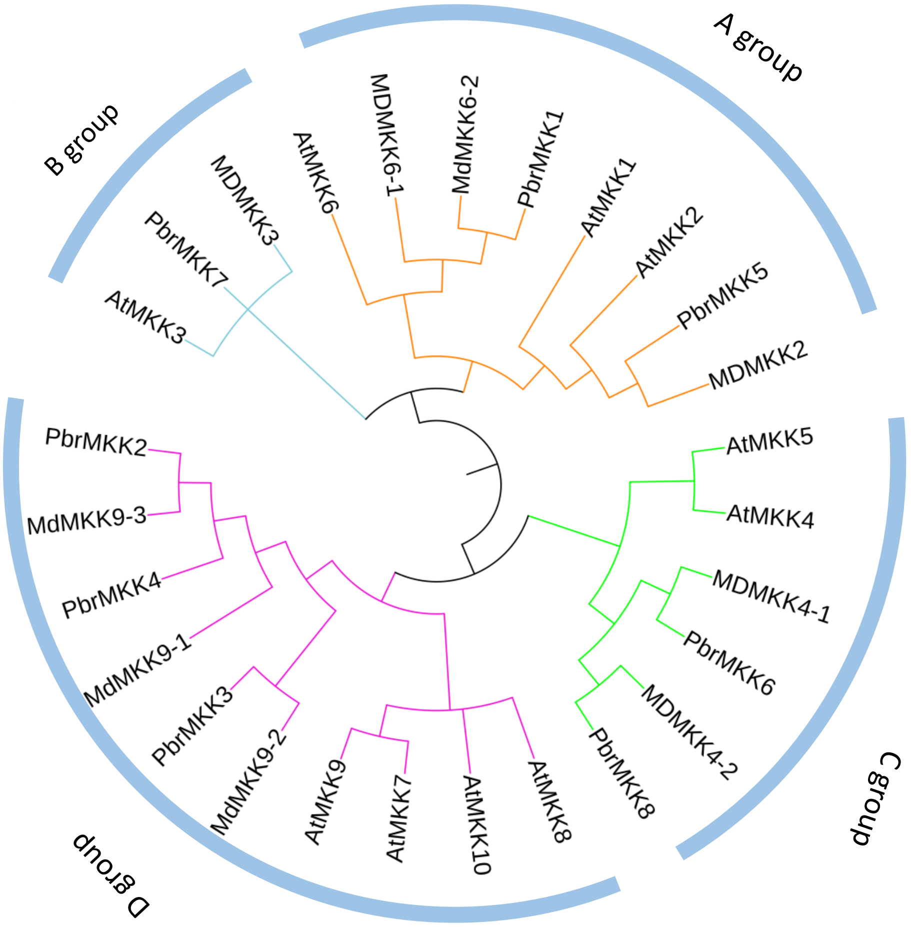

Figure S1. Phylogenetic analysis of MAPKKs from *Pyrus*, *Malus*, *Arabidopsis*. The Neighbor-joining (NJ) tree was constructed using ClustalX 1.8 and MEGAX32 software.

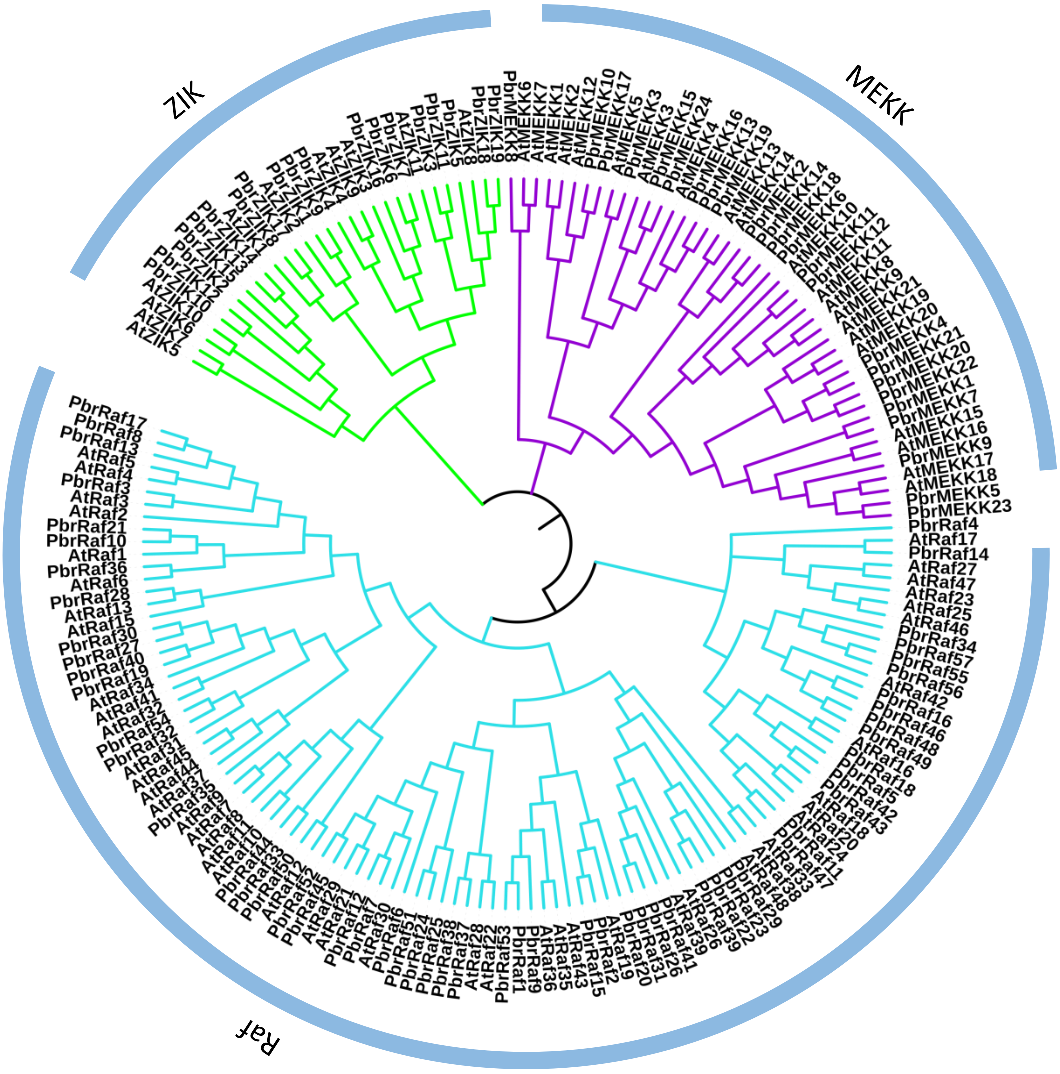

Figure S2. Phylogenetic analysis of MAPKKKs from *Pyrus* and *Arabidopsis*. The Neighbor-joining (NJ) tree was constructed using ClustalX 1.8 and MEGAX32 software.

MAPKK

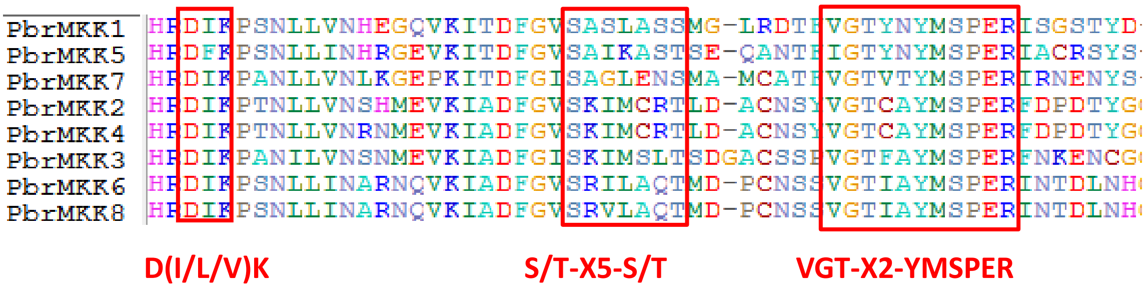

Figure S3. Multiple sequence alignment analysis of the peptides of MAPKK in *Pyrus*. The highlighted part showed the conserved signature motif obtained with the ClustalX program.

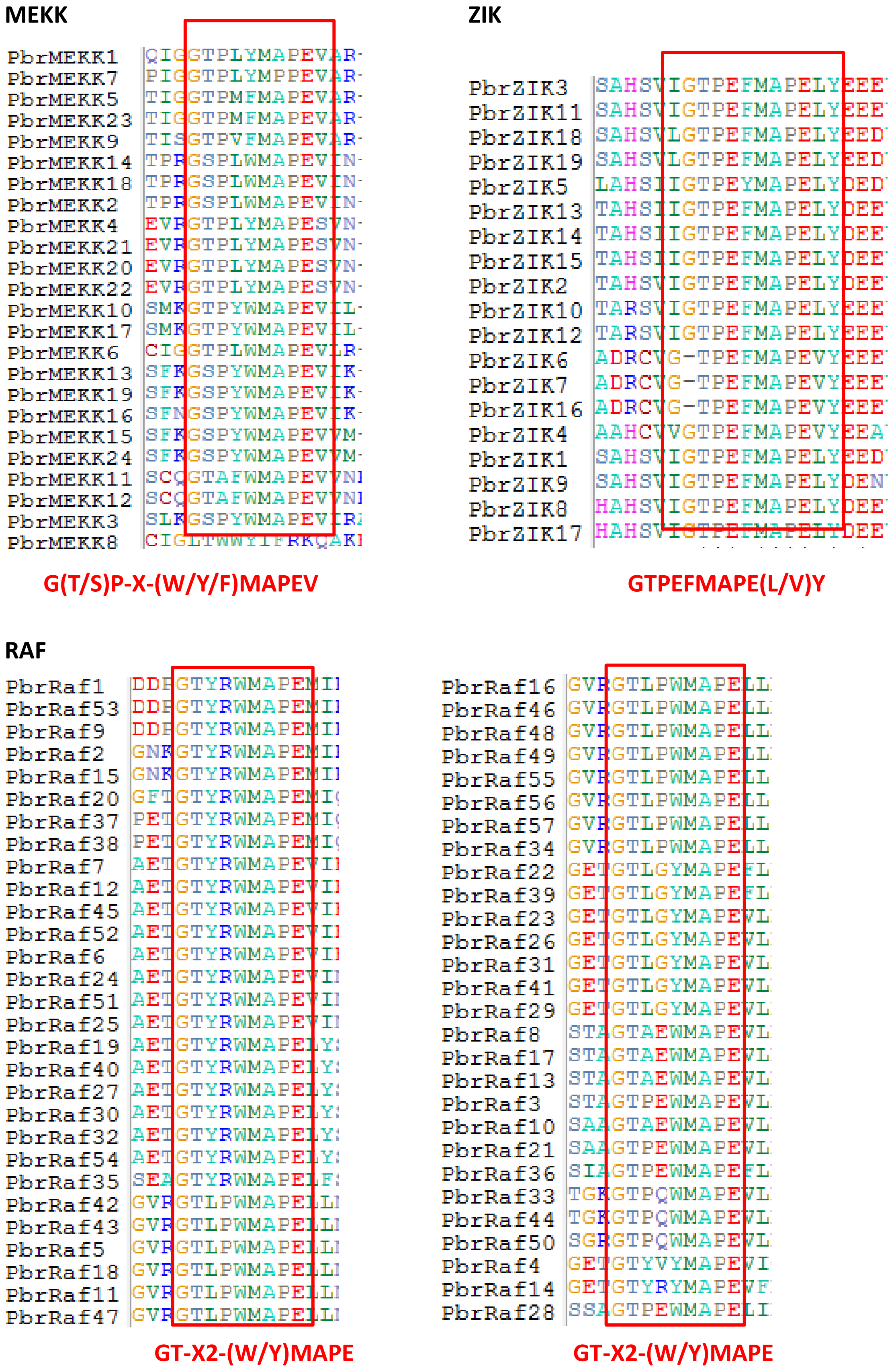

Figure S4. Multiple sequence alignment analysis of the peptides of MAPKKK proteins in *Pyrus*. The highlighted part showed the conserved signature motif obtained with the ClustalX program.

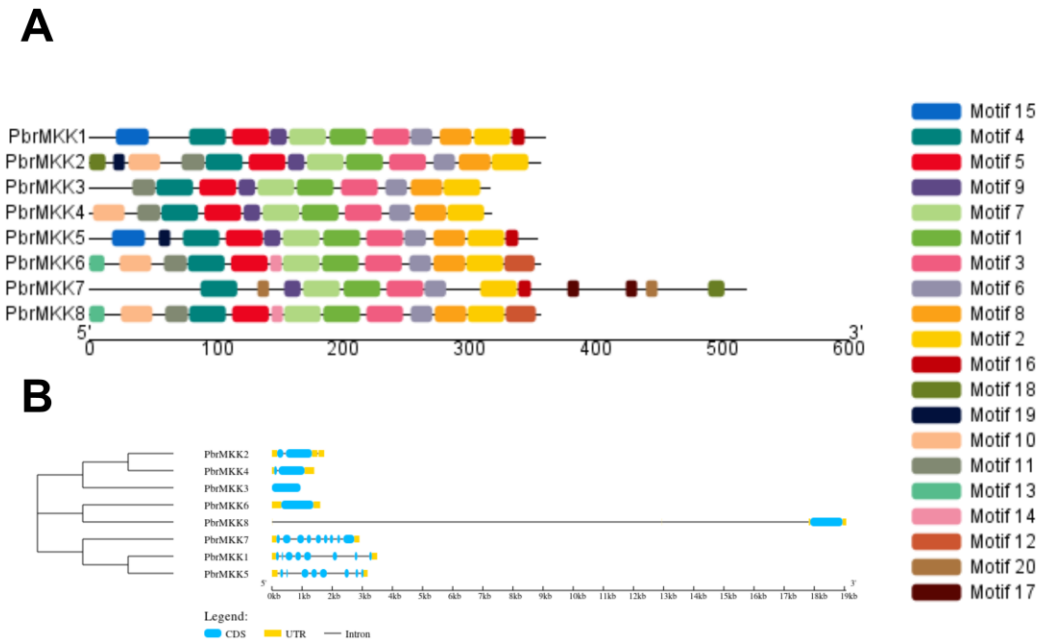

Figure S5. (**A**) Conserved motifs of PbrMAPKKs. All motifs were identified by MEME database. (**B**) Phylogenetic relationship and exon/intron structure of PbrMAPKKs. Gene structures were drawn using GSDS database. The blue boxes, green boxes, and the black lines indicate upstream/downstream, exons and introns, respectively.

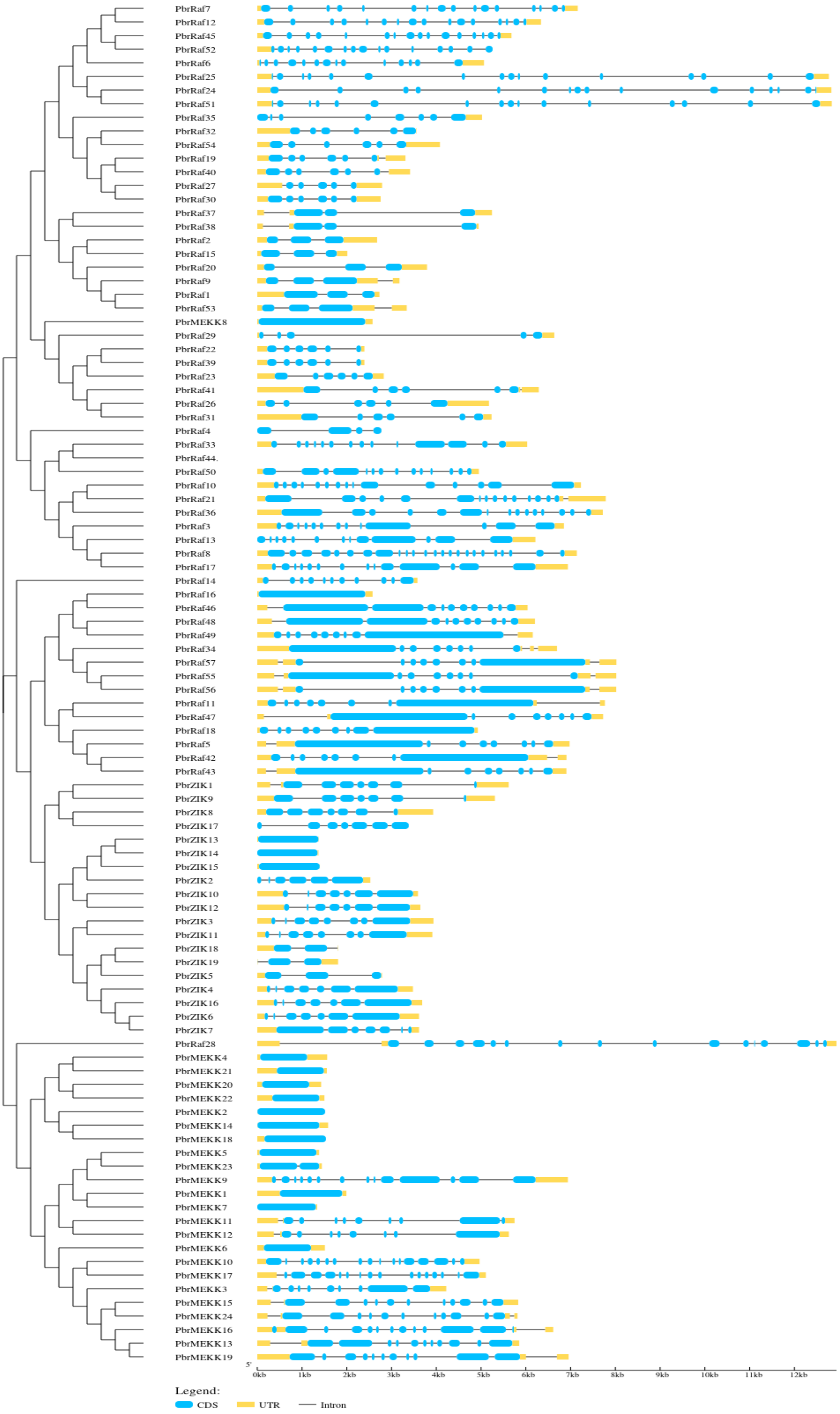

Figure S6. Phylogenetic relationship and exon/intron structure of PbrMAPKKs in *Pyrus*. For other details, see Figure S5.

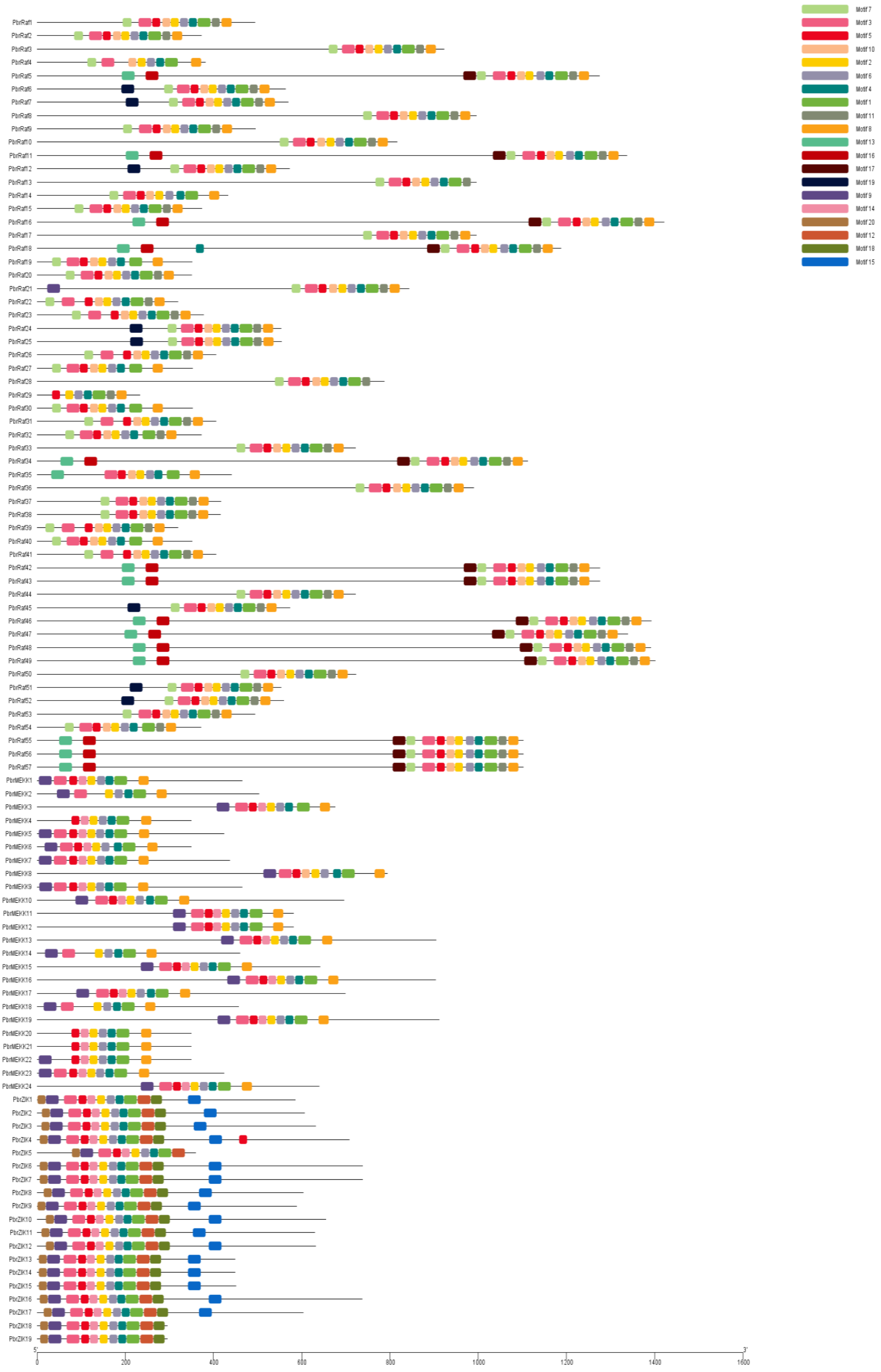

Figure S7. Conserved motifs of PbrMAPKKK family. All motifs were identified by MEME database.
