# Supplementary material for: Genome-Wide Identification of MAPKK and MAPKKK Gene Family Members and Transcriptional Profiling Analysis during Bud Dormancy in Pear (Pyrus x bretschneideri)
Source: Plants (Basel). 2022 Jun 29;11(13):1731. doi: 10.3390/plants11131731 (PMC9269224; doi:10.3390/plants11131731)
Supplement: Supplementary file 1 [file plants-11-01731-s001.zip › Supplementary Tables.pdf]

Table S1. Sequences of primers used in qRT-PCR**.**

Target Gene	Forward primer sequence (5′→3′)	Reverse primer sequence (5′→3)
PbrMAPK3	AGACCTCGTTCCCGCTAA	ATCGCCACCATCTCCTTC
PbrMAPK5	ACTGTTGAAGATGCCCT	ACTGTAGCAGATACTCGGG
PbrMAPK20	CAAAGAGGATTAGTGTCGCTGA	CCACATCATCTCCCTTATCGT
PbrMKK6	AACCCAGCAGTGGTG	GCTCGGAGAAGTTGATG
PbrMKK8	CACCACCGAGACAAGTA	CGGAGAAGTTGATGAGC
PbrMEKK1	TATCGCCGCCAAATGAG	GCAAGCCTTTCTCTTAGTATGC
PbrMEKK7	TTCACCTCCTACCCG	GCTCTTCCTATGTTTACTGC
PbrMEKK12	TGAAGAGACTGTATCGGTC	TACCAAGAAGGTCACCC
PpActin	CCATCCAGGCTGTTCTCTC	GCAAGGTCCAGACGAAGG

Table S2. Characteristics of the MAPKK genes in *Pyrus*. GeneID is accession number in NCBI database. Length (number of amino acids), molecular weight (kilodaltons), and isoelectric point (pI) of the deduced polypeptides were calculated using ExPASy (http://web.expasy.org/protparam/).

Name	GeneID	Gene model	Chromosomes Position			Lenth	Size	MW	PI
			Chr	Chr_start	Chr_end	(bp)	(aa)	(KD)	
PbrMKK1	103943916	XM_009354287.2	chr15	12807770	12811252	1392	360	40.4	5.66
PbrMKK2	103946907	XM_009357731.2	chr6	19995653	19996924	1613	356	39.9	7.6
PbrMKK3	103948714	XM_009359774.1	chr16	9952473	9953423	951	316	34.8	7.18
PbrMKK4	103950550	XM_018648269.1	chr14	18547654	18549047	1349	318	35.9	8.01
PbrMKK5	103951897	XM_009363380.2	chr2	3964839	3968053	1393	354	39.6	5.93
PbrMKK6	103926835	XM_009335718.2	chr9	13887479	13889041	1603	356	39.6	9.3
PbrMKK7	103926960	XM_009335845.2	chr9	19221509	19224402	1872	519	57.8	5.52
PbrMKK8	103931740	XM_009341260.1	chr17	11264564	11283625	1252	357	39.4	9.15

Table S3. Characteristics of the MAPKKK genes in *Pyrus*. Genes have not been identified on chromosomes are presented scaffold sites.

Name	GeneID	Gene model	Chromosomes Position			Lenth	Size	MW	PI
			Chr	Chr_start	Chr_end	(bp)	(aa)	(KD)	
PbrMEKK1	103943998	XM_009354390.2	chr5	26148621	26150210	1992	465	51.0	5.17
PbrMEKK2	103948844	XM_009359922.2	chr3	25835450	25836969	1517	503	55.8	6.34
PbrMEKK3	103953927	XM_009365739.2	chr15	2567864	2572048	2626	676	73.0	9.58
PbrMEKK4	103956878	XM_009368934.2	chr2	9230941	9232307	1563	350	39.0	5.33

PbrMEKK5	103944552	XM_009355024.2	chr9	15956010	15957392	1383	424	46.6	4.58
PbrMEKK6	103946463	XM_009357171.2	chr8	1048846	1049898	1512	350	38.2	5.29
PbrMEKK7	103948309	XM_009359316.2	chr10	24661825	24663138	1339	437	48.5	6.03
PbrMEKK8	103949475	XM_009360594.2	chr12	14416576	14419100	2578	794	89.0	5.72
PbrMEKK9	103951455	XM_009362823.2	chr3	5822942	5824343	1473	465	51.6	4.83
PbrMEKK10	103954127	XM_009365929.2	chr13	10053870	10058754	2622	696	76.7	7.57
PbrMEKK11	103956703	XM_009368727.2	chr15	33073337	33079077	2441	581	63.8	5.74
PbrMEKK12	103956986	XM_009369043.2	chr8	5584969	5590579	2356	581	63.9	5.48
PbrMEKK13	103958240	XM_009370489.2	chr7	21211337	21217170	3294	904	97.4	9.46
PbrMEKK14	103959029	XM_009371364.2	chr11	4030271	4031757	1584	460	50.1	4.9
PbrMEKK15	103960739	XM_009373217.2	chr11	8169044	8174880	2580	642	70.1	9.03
PbrMEKK16	103963863	XM_009376720.2	chr6	8961864	8968465	3574	903	98.7	9.02
PbrMEKK17	103930807	XM_009340185.2	chr16	4892659	4897765	2581	699	76.9	6.76
PbrMEKK18	103931255	XM_009340689.2	chr11	5208835	5210359	1536	457	49.9	5.02
PbrMEKK19	103932227	XM_009341784.2	chr1	894205	901153	3852	911	98.3	9.3
PbrMEKK20	103935136	XM_009344894.2	chr15	27626196	27627541	1428	350	38.9	4.91
PbrMEKK21	103935253	XM_009345019.2	NW_008988737.1(198540..200095)			1556	350	38.7	5.25
PbrMEKK22	103937739	XM_009347685.2	chr15	20605457	20606771	1501	350	39.0	4.96
PbrMEKK23	103938810	XM_009348854.2	NW_008989007.1 (48323..49766)			1389	424	46.7	4.61
PbrMEKK24	103938937	XM_009348988.2	chr3	20379870	20385507	2370	640	69.8	9.15
PbrRaf1	103932587	XM_009342251.2	chr5	24303758	24306476	2191	494	55.5	9.46
PbrRaf2	103946226	XM_009356910.2	chr5	26417025	26419613	2088	373	42.4	9.14
PbrRaf3	103952008	XM_009363533.2	chr15	3875425	3882271	3410	923	102	5.36
PbrRaf4	103955468	XM_018649421.1	chr15	3461007	3464817	1149	382	43.3	5.3
PbrRaf5	103960467	XM_009372957.2	chr5	14948136	14955110	4802	1275	141	5.49
PbrRaf6	103927283	XM_009336190.1	chr15	218901	224813	2358	563	62.9	6.54
PbrRaf7	103939525	XM_009349625.2	chr6	19338293	19345443	2079	569	64.1	6.28
PbrRaf8	103944043	XM_009354433.2	chr10	23756445	23763490	3539	1010	112.1	5.31
PbrRaf9	103944202	XM_009354635.2	chr10	22543921	22547097	2291	495	55.7	9.44
PbrRaf10	103944303	XM_009354727.1	chr14	288298	295530	2974	816	90.6	6.15
PbrRaf11	103945170	XM_009355705.2	chr10	20086142	20093905	4433	1337	147.7	5.36
PbrRaf12	103946586	XM_009357311.2	chr11	23485718	23491913	2195	572	63.9	5.87
PbrRaf13	103946949	XM_018647410.1	chr6	20257729	20264497	3500	996	109.1	5.59
PbrRaf14	103947202	XM_009358061.2	chr13	2736990	2740574	1507	433	48.9	6.67
PbrRaf15	103948242	XM_009359252.2	chr10	24937897	24939874	1445	374	42.7	9.17
PbrRaf16	103949135	XM_009360237.2	chr15	10379172	10385264	4894	1421	154.4	5.35
PbrRaf17	103950670	XM_009361900.2	chr14	18245153	18252081	4040	996	109.6	5.48
PbrRaf18	103952248	XM_009363804.2	chr13	14132133	14136931	3694	1187	132.8	5.9
PbrRaf19	103952951	XM_009364664.2	chr14	18795442	18798648	1791	352	39.9	7.64
PbrRaf20	103953036	XM_009364756.2	chr14	19563346	19566565	1768	351	39.9	8.32
PbrRaf21	103955172	XM_009367049.2	chr12	1341933	1349708	3635	843	93.0	5.75
PbrRaf22	103956904	XM_009368961.2	chr17	4538394	4540793	1256	319	35.7	8.17
PbrRaf23	103957204	XM_009369321.2	chr11	29400163	29402980	1772	378	42.2	8.17
PbrRaf24	103957768	XM_009369972.1	chr10	791535	804362	2288	553	62.1	5.05

PbrRaf25	103957984	XM_009370209.2	chr5	1537126	1549885	2323	554	62.3	5.19
PbrRaf26	103959250	XM_009371602.2	chr16	6851265	6855967	2334	406	45.3	7.06
PbrRaf27	103959717	XM_009372080.2	chr17	21115632	21118406	1871	353	39.6	7.65
PbrRaf28	103959884	XM_009372253.2	chr17	2925891	2938686	3226	787	88.7	5.95
PbrRaf29	103960105	XM_009372518.2	chr3	1882328	1888921	1012	233	26.7	9.21
PbrRaf30	103960980	XM_009373485.2	chr17	22672905	22675632	1839	353	39.6	7.65
PbrRaf31	103961155	XM_009373685.2	chr16	7887459	7891758	2391	406	45.3	7.06
PbrRaf32	103961197	XM_009373730.2	chr12	16513839	16517633	1865	373	41.8	9.03
PbrRaf33	103961300	XM_018650921.1	chr13	7023446	7029476	2956	722	80.8	6.00
PbrRaf34	103962396	XM_018651181.1	chr13	18211137	18217816	4590	1112	123.8	5.47
PbrRaf35	103963188	XM_018651399.1	chr15	17541829	17546850	1685	441	49.7	7.16
PbrRaf36	103964928	XM_009377926.2	chr8	4181678	4188925	3777	990	110.8	6.43
PbrRaf37	103965044	XM_018651847.1	chr4	22148950	22154196	1882	417	47.0	6.64
PbrRaf38	103965043	XM_018651848.1	chr4	22155018	22160008	1529	416	46.7	6.51
PbrRaf39	103967891	XM_009381221.2	chr11	1837205	1839604	1256	319	35.7	8.17
PbrRaf40	103926790	XM_009335667.2	chr6	15897077	15900481	1729	352	39.8	6.61
PbrRaf41	103928016	XM_018642764.1	chr13	10679794	10686052	2672	406	45.3	7.03
PbrRaf42	103928684	XM_009337771.2	chr10	14817267	14824162	4755	1276	140.8	5.82
PbrRaf43	103928702	XM_009337777.2	chr10	14904417	14911313	4753	1276	140.8	5.82
PbrRaf44	103928908	XM_018642931.1	chr17	6948261	6954075	2748	722	80.9	6.00
PbrRaf45	103929586	XM_009338810.2	chr15	13838396	13844083	2092	573	64.7	5.76
PbrRaf46	103930770	XM_009340142.2	NW_008988522.1(232419..238458)			4669	1392	150.8	5.37
PbrRaf47	103932432	XM_018643775.1	chr10	18828569	18829558	4500	1339	147.8	5.29
PbrRaf48	103933869	XM_009343567.2	chr2	1868848	1875047	4877	1391	151.1	5.29
PbrRaf49	103933866	XM_009343564.2	chr2	2107148	2113294	4913	1401	152.2	5.27
PbrRaf50	103934596	XM_009344349.2	chr4	16468479	16473426	2459	723	81.4	6.35
PbrRaf51	103935569	XM_009345337.2	chr10	937205	950035	2248	553	62.1	5.05
PbrRaf52	103936707	XM_009346559.1	chr2	6521142	6526400	1993	559	63.1	5.82
PbrRaf53	103938439	XM_009348446.2	NW_008988975.1(130200..133539)			2414	494	55.5	9.46
PbrRaf54	103938616	XM_009348640.2	chr4	1653399	1657472	2154	372	41.8	8.90
PbrRaf55	103939837	XM_009349946.2	NW_008989102.1 (27943..35963)			4533	1102	122.0	5.41
PbrRaf56	103939838	XM_009349948.2	NW_008989102.1 (84446..92465)			4532	1102	122.0	5.41
PbrRaf57	103940409	XM_009350526.2	chr16	11736005	11743972	4534	1102	121.9	5.43
PbrZIK1	103945882	XM_009356477.2	chr3	25032591	25038209	2813	585	66.2	5.89
PbrZIK2	103949289	XM_009360398.2	chr6	12601430	12604452	1983	606	67.7	5.28
PbrZIK3	103952945	XM_009364654.2	chr14	18758387	18762261	2735	632	70.7	4.87
PbrZIK4	103954434	XM_009366267.2	chr12	19872179	19875646	2677	708	80.0	5.42
PbrZIK5	103955052	XM_009366921.2	chr9	14418639	14421411	1271	360	40.6	7.17
PbrZIK6	103956633	XM_009368661.2	chr9	19533807	19537354	2812	738	83.9	5.24
PbrZIK7	103956637	XM_009368668.2	chr9	19487037	19490584	2812	738	83.9	5.24
PbrZIK8	103961194	XM_018650886.1	chr12	16435306	16439194	2795	603	68.4	5.38
PbrZIK9	103963292	XM_009376089.2	chr11	3255734	3261041	2775	588	66.5	6.25
PbrZIK10	103964241	XM_009377151.2	chr1	9564975	9568068	2646	655	73.2	5.43
PbrZIK11	103926788	XM_009335664.2	chr6	15941855	15945831	2642	630	70.7	4.89

PbrZIK12	103927211	XM_018642556.1	chr7	12526084	12528903	2726	632	70.8	5.5
PbrZIK13	103933162	XM_009342828.2	NW_008988626.1(239361..240736)			1376	449	51.0	5.21
PbrZIK14	103933153	XM_009342818.1	NW_008988626.1 (36926..38294)			1369	449	51.0	5.21
PbrZIK15	103935873	XM_009345688.2	chr13	11496461	11497835	1400	451	51.3	5.16
PbrZIK16	103935985	XM_009345820.2	chr13	10332029	10335696	2819	737	83.8	5.47
PbrZIK17	103938621	XM_009348648.2	chr4	1586583	1590765	1812	603	68.8	5.82
PbrZIK18	103941126	XM_009351316.1	NW_008989269.1 (16258..18212)			1267	295	33.8	5.49
PbrZIK19	103941128	XM_009351318.1	NW_008989269.1 (71964..73918)			1267	295	33.8	5.49

Table S4. Analysis of variance of 8 MAPK cascade genes response to 1%HC treatment at 0 day, 0.5 day, 3 day, and 9 day.

Gene	treat date		HC		ANOVA		HCK		ANOVA
PbrMAPK3	0d	1.16	0.98	0.88	0.01	1.16	0.98	0.88	0.01
	0.5d	0.86	0.8	0.68	0.01	1.43	0.87	1.18	0.05
	3d	654.08	194.46	663.22	47896.67	8.67	9.11	8.21	0.14
	9d	405.44	125.66	185.25	14479.05	183.55	182.28	250.73	1022.24
PbrMAPK5	0d	0.95	0.91	1.16	0.01	0.95	0.91	1.16	0.01
	0.5d	0.13	0.09	0.09	0	0.11	0.07	0.09	0
	3d	0.67	0.79	0.76	0	0.85	0.52	0.56	0.02
	9d	0.43	0.55	0.3	0.01	0.23	0.23	0.26	0
PbrMAPK20	0d	1.1	1.36	0.67	0.08	1.1	1.36	0.67	0.08
	0.5d	0.45	0.51	0.42	0	0.49	0.52	0.41	0
	3d	1.88	1.71	1.61	0.01	1.21	1.18	1.62	0.04
	9d	0.71	0.78	1.01	0.02	1.42	1.41	1.85	0.04
PbrMKK6	0d	0.59	2.53	0.66	0.81	0.59	2.53	0.66	0.81
	0.5d	1.95	2.91	1.48	0.35	1.97	1.87	1.63	0.02
	3d	23.86	25.75	26.85	1.52	1.34	1.82	2.2	0.12
	9d	11.37	12.35	9.76	1.14	10.36	9.27	14.96	6.08
PbrMKK8	0d	1.29	1.07	0.72	0.06	1.29	1.07	0.72	0.06
	0.5d	3.78	4.37	3.33	0.18	5.5	3.18	4.11	0.91
	3d	48.17	38.05	52.71	37.55	7.91	7.48	9.34	0.63
	9d	39.12	41.64	32.67	14.27	49.41	30.63	52.59	93.89
PbrMEKK1	0d	0.91	0.93	1.19	0.02	0.91	0.93	1.19	0.02
	0.5d	2.1	2.13	2.4	0.02	4.78	4.22	4.34	0.06
	3d	54.07	68.91	71.84	60.51	14.12	14.22	16.34	1.05
	9d	37.7	31.27	30.84	9.84	44.74	52.47	49.29	10.06
PbrMEKK7	0d	1.02	1.02	0.97	0	1.02	1.02	0.97	0
	0.5d	0.07	0.04	0.03	0	0.16	0.18	0.14	0
	3d	8.75	18.25	12.64	15.21	7.33	14.25	10.58	7.99
	9d	59.71	44.32	53.82	40.2	38.5	36.67	34.7	2.41
PbrMEKK12	0d	1.15	0.82	1.05	0.02	1.15	0.82	1.05	0.02
	0.5d	0.29	0.24	0.38	0	0.35	0.34	0.4	0
	3d	6.03	6.2	8.13	0.91	3.2	2.98	4.4	0.39
	9d	2.37	2.89	4.77	1.06	5.13	4.35	3.32	0.55

Table S5. Analysis of variance of 8 MAPK cascade genes response to ABA treatment at 0 day, 0.5 day, 3 day, and 9 day.

Gene	treat date		ABA		ANOVA		ACK		ANOVA
PbrMAPK3	0d	1.24	0.93	0.87	0.03	1.24	0.93	0.87	0.03
	0.5d	0.01	0.01	0.01	0	1.31	1.21	1.02	0.01
	3d	0.09	0.23	0.17	0	0.07	0.21	0.04	0.01
	9d	0.22	0.71	0.32	0.04	0.17	0.13	0.15	0
PbrMAPK5	0d	9.17	9.23	9.56	0.03	9.17	9.23	9.56	0.03
	0.5d	0.05	0.05	0.04	0	2.03	3.26	2.93	0.27
	3d	2.97	1.93	2.41	0.18	0.87	1.17	1.43	0.05
	9d	3.56	4.51	3.74	0.17	0.52	0.5	0.33	0.01
PbrMAPK20	0d	6.76	6.67	6.9	0.01	6.76	6.67	6.9	0.01
	0.5d	0.03	0.04	0.04	0	1.76	2.15	2.37	0.06
	3d	1.26	1.09	1.19	0	1.48	1.45	1.33	0
	9d	1.65	1.62	1.29	0.03	0.42	0.42	0.52	0
PbrMKK6	0d	0.91	1.12	0.98	0.01	0.91	1.12	0.98	0.01
	0.5d	0	0	0	0	0.26	0.27	0.3	0
	3d	0.04	0.04	0.04	0	0.05	0.04	0.04	0
	9d	0.11	0.11	0.16	0	0.03	0.03	0.03	0
PbrMKK8	0d	1.02	1	0.98	0	1.02	1	0.98	0
	0.5d	0.03	0.03	0.02	0	0.47	0.49	0.79	0.02
	3d	0.04	0.06	0.03	0	0.02	0.01	0.02	0
	9d	0.2	0.24	0.3	0	0.04	0.03	0.06	0
PbrMEKK1	0d	0.95	0.99	1.06	0	0.95	0.99	1.06	0
	0.5d	0	0	0	0	0.28	0.37	0.59	0.02
	3d	0.01	0.02	0.02	0	0.02	0.02	0.03	0
	9d	0.13	0.11	0.24	0	0.06	0.03	0.05	0
PbrMEKK7	0d	1.78	0.78	0.72	0.24	1.78	0.78	0.72	0.24
	0.5d	0	0	0	0	0.24	0.24	0.26	0
	3d	0.13	0.14	0.13	0	0.1	0.1	0.1	0
	9d	0.24	0.33	0.41	0	0.07	0.06	0.08	0
PbrMEKK12	0d	1.09	1.06	0.86	0.01	1.09	1.06	0.86	0.01
	0.5d	0.03	0.07	0.05	0	0.33	0.32	0.34	0
	3d	0.04	0.03	0.04	0	0.05	0.14	0.1	0
	9d	0.26	0.46	0.36	0.01	0.16	0.12	0.15	0
